# Metabolic Responses of *Pyropia haitanensis* to Dehydration-Rehydration Cycles Revealed by Metabolomics

**DOI:** 10.3390/md23050203

**Published:** 2025-05-08

**Authors:** Jian Wen, Jianzhi Shi, Muhan Meng, Kai Xu, Yan Xu, Dehua Ji, Wenlei Wang, Chaotian Xie

**Affiliations:** 1 Fisheries College, Jimei University, Xiamen 361021, China; 202214908003@jmu.edu.cn (J.W.); shijianzhi1989@163.com (J.S.); 15222331526@163.com (M.M.); kaixu@jmu.edu.cn (K.X.); yanxu@jmu.edu.cn (Y.X.); dhji@jmu.edu.cn (D.J.); 2 Fujian Engineering Research Center of Aquatic Breeding and Healthy Aquaculture, Xiamen 361021, China; 3 State Key Laboratory of Mariculture Breeding, Fisheries College, Jimei University, Ningde 352100, China

**Keywords:** *Pyropia haitanensis*, desiccation tolerance, osmotic adjustment, antioxidant system, plant hormones, rehydration, LC-MS based metabolomics

## Abstract

*Pyropia haitanensis* (T.J. Chang and B.F. Zheng) undergoes periodic dehydration and rehydration cycles, necessitating robust adaptive mechanisms. Despite extensive research on its physiological responses to desiccation stress, the comprehensive metabolic pathways and recovery mechanisms post-rehydration remain poorly understood. This study investigated the metabolic responses of *P. haitanensis* to varying degrees of desiccation stress using LC-MS and UPLC-MS/MS. Under mild dehydration, the thallus primarily accumulated sugars and proline, while moderate and severe dehydration triggered the accumulation of additional osmoprotectants like alanine betaine and trehalose to maintain turgor pressure and water retention. Concurrently, the alga activated a potent antioxidant system, including enzymes and non-enzymatic antioxidants, to counteract the increased reactive oxygen species levels and prevent oxidative damage. Hormonal regulation also plays a crucial role in stress adaptation, with salicylic acid and jasmonic acid upregulating under mild dehydration and cytokinins and gibberellin GA_15_ accumulating under severe stress. Rehydration triggered the recovery process, with indole acetic acid, abscisic acid, and jasmonic acid promoting rapid cell recovery. Additionally, arachidonic acid, acting as a signaling molecule, induced general stress resistance, facilitating the adaptation of the thallus to the dynamic intertidal environment. These findings reveal *P. haitanensis*’ metabolic adaptation strategies in intertidal environments, with implications for enhancing cultivation and stress resistance in this economically important seaweed.

## 1. Introduction

Intertidal macroalgae inhabit an inherently stressful environment, where periods of immersion in seawater alternate with aerial exposure as periodic tidal rhythms. It experiences severe desiccation stress during low tides [1,2,3]. Fortunately, these macroalga have developed unique mechanisms to cope with severe desiccation challenge and to recover to normal physiological activity after tides rise. Additionally, the intertidal zone is a transition region where organisms evolved from oceanic to terrestrial life [4,5]. According to reports, red algae diverged from green algae more than 1 billion years ago [6]. Thus, intertidal macroalgae are considered to be ideal biological model to investigate stress ecophysiology of seaweed communities but also of higher land plants [1,2,7,8,9].

*Pyropia* spp. is a typical representative of large red algae in the intertidal zone [2]. It is not only an important economic species in the global algae industry, but also a key marine resource with both ecological value and nutritional functions. Over 130 species have been documented worldwide, with *Pyropia yezoensis* and *Pyropia haitanensis* being the primary species cultivated in aquaculture [10]. According to 2022 data from the Food and Agriculture Organization (FAO) of the United Nations, its global annual output has reached 2.96 million tons (wet weight) [11], ranking among the top in economic seaweed production. In recent years, with the rapid development of the global algae industry, seaweed applications have become increasingly diverse, expanding beyond traditional fields such as food, aquaculture feed, and fertilizers to encompass emerging areas like pharmaceuticals and ecological restoration [12,13,14]. *Pyropia* often undergoes desiccation stresses in its whole life. For example, *Pyropia* can lose more than 90% of water when exposed to air during low tide [15]. The farmers often raised them out of the sea in order to kill diatoms, invertebrate larvae and others fouling organisms in the nets seeded with *Pyropia* thalli [16]. Surprisingly, the physiological activity of *Pyropia* would recover rapidly when re-immersed in seawater, and the resistance of *Pyropia* to disease was also strengthened, which improve the production and umami of *Pyropia*. The farming protocols with and without periodic desiccation affects dehydration tolerance and nutrient components of *Pyropia* [17].

Several studies have investigated the mechanisms underlying *Pyropia*’s exceptional desiccation tolerance. For example, Wang et al., 2015 conducted a transcriptomic analysis of *P. haitanensis* under desiccation stress [18]. Their findings revealed that multiple biological processes, including chlorophyll biosynthesis, apoptosis regulation, and ABC transporter activity, play crucial roles in osmotic stress response [18]. Im et al., 2017 identified the *PtDRG2* from *Pyropia tenera* based on the transcriptome analysis, and revealed that *PtDRG2* conferred osmotic and salt tolerance in transgenic *Chlamydomonas* cells [19]. Additionally, the differently expressed proteins involved in the tolerance of *Pyropia orbicularis*, including decreased photosynthetic rate, increased antioxidant activity, and the preservation of cell physiology, are activated during low tide [20]. Yin et al., 2025 discovered that under dehydration stress, second messengers such as reactive oxygen species (ROS) and Ca^2+^ signals play a crucial role [21]. These messengers interact with actin and actin-binding proteins, thereby influencing or regulating the dynamics and reorganization of microfilaments in *P. yezoensis*. These dynamic changes in the cytoskeleton have far-reaching consequences. They trigger alterations in various intracellular activities within *P. yezoensis*. For instance, the activity of transcription factors, photosynthesis, and CO_2_ fixation are all affected [21]. In a separate study, Xu et al., 2016 investigated the response mechanisms of *P. haitanensis* to dehydration stress [22]. They identified 100 differentially expressed protein spots in this species, with the largest protein grouping related to photosynthesis and energy metabolism [22]. Meanwhile, in our previous study, we screened transketolase of *P. haitanensis* based on integrative transcriptome and proteomic analyses, which could improve the resistance of *Chlamydomonas* to osmotic stress [23]. However, although the advancement in transcriptomics and proteomics for *Pyropia* in recent times, the genetic mechanisms regulating the various biochemical pathways still remain largely unexplored.

Metabolomics, a powerful platform for the global low-molecular-weight metabolites identification and quantification in plants, provides high-resolution snapshot of various cell’s catalytic and regulatory metabolic processes related to plant and environment interactions [24,25]. It presents the information of biological relevance as it reflects the immediate biochemical consequences of genomic and transcriptomic activity [26]. Ye et al., 2014 determined the nutrient composition of *P. yezoensis*, which was dominated by 11 carboxylic acids, 11 amino acids, four sugars, and four choline metabolites by using the technologies of nuclear magnetic resonance [27]. Liu et al., 2025 employed metabolomics technology to analyze the metabolic changes throughout the development of *P. haitanensis* conchosporangia, revealing that the lipoxygenase pathway may be involved in the formation of conchosporangia [28]. Their study showed that during maturation, C18 and C20 derived oxylipins, including oxo-eicosatetraenoic acid and prostaglandins, increased significantly [28]. In a separate study using non-targeted gas chromatography-mass spectrometry (GC-MS, Agilent J and W Scientific, USA), Jian et al., 2017 demonstrated that 1-octen-3-ol promotes *P. haitanensis* cell growth by regulating primary metabolism (e.g., glycerol-3-phosphate and organic acids) under temperature stress [29].

While metabolomics has been extensively applied to study plant-environment interactions, further research is needed to fully elucidate the metabolic responses of *P. haitanensis* to dehydration stress. Chen et al., 2022 conducted a comprehensive multi-omics association analysis employing genomics, metabolomics, and other methodologies, revealing that under dehydration stress, *P. haitanensis* mitigates light damage by decreasing the content of light-harvesting pigments [5]. Simultaneously, it enhances the synthesis of glutathione (GSH) and ascorbic acid to alleviate oxidative stress. Furthermore, the downregulation of genes and metabolites associated with the Calvin cycle indicates that the thalli may respond to drought stress by reducing energy metabolism [5]. While this research provides valuable insights into the metabolic response of *P. haitanensis* during dehydration, it primarily focuses on dehydration stress, with limited exploration of recovery mechanisms after rehydration. Additionally, although the study included analysis of free polyunsaturated fatty acids and membrane lipids, a more comprehensive verification of key metabolites and their dynamic changes during both dehydration and rehydration processes is still needed. Therefore, further exploration and enrichment are still required to comprehend the essential metabolic pathways and metabolites involved in the response of *P. haitanensis* to dehydration and rehydration processes. In the present study, we investigated metabolic changes in *P. haitanensis* under desiccation stress (0%, 30%, 60%, 80%, and Rehydration) using untargeted liquid chromatography-mass spectrometry (LC-MS) for global metabolite profiling, followed by targeted ultra-performance liquid chromatography-tandem mass spectrometry (UPLC-MS/MS) for validation of key metabolites. The present results will be of great importance for comprehending the desiccation tolerance behind intertidal macroalgae.

## 2. Results

### 2.1. Evaluation of Metabolomics Reproducibility

**Figure 1:**
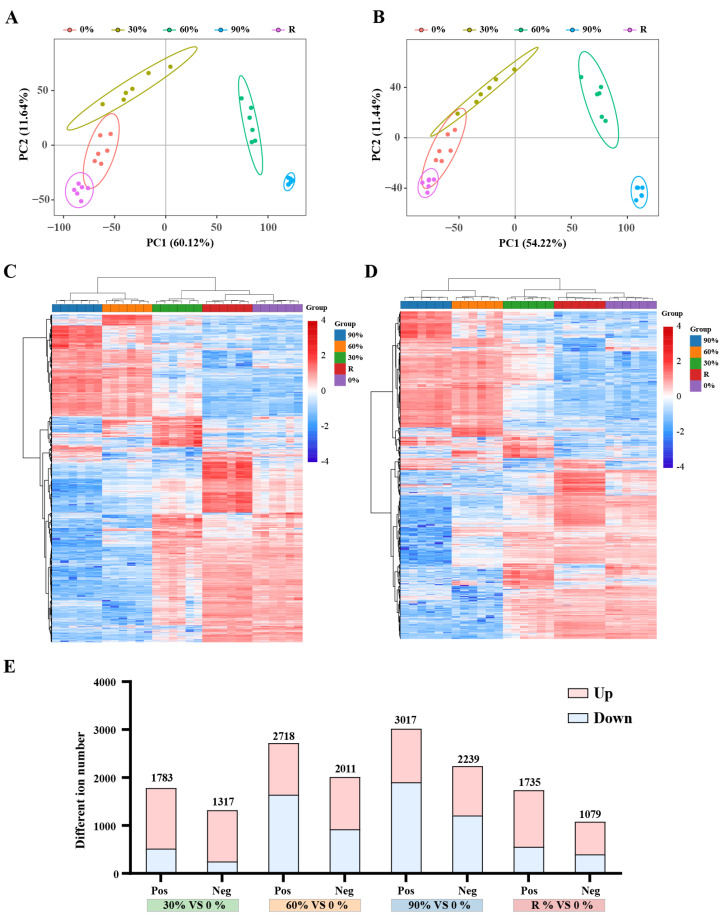
Statistical analysis of metabolome information of *P. haitanensis* after different dehydration stress conditions. (**A**) Principal component analysis (PCA) of metabolite patterns under different dehydration stress conditions in positive ion mode. (**B**) PCA of metabolite patterns under different dehydration stress conditions in negative ion mode. Numbers in parentheses represent the percentage of the total variance explained by the first and second principal components (PC). Symbols of the same color represent the biological replicates for each treatment. 0%, control; 30%, 60%, and 90% represent the corresponding water loss rates; R, recovery. (**C**) Clustering analysis of ions in positive ion mode. (**D**) Clustering analysis of ions in negative ion mode. (**E**) The number of differentially expressed ions in the *P. haitanensis* after different levels of dehydration treatment. VIP ≥ 1, |Fold Change| ≥ 1.2 and *q*-value < 0.05.

For the data extracted from the metabolome, the present study initially conducted missing value imputation and removal of low-quality ions (ions missing in more than 50% of QC samples or in more than 80% of actual samples were removed). Subsequently, data filtering was performed with the criterion of a relative standard deviation >30% in QC samples, ultimately yielding 7975 and 5401 positive and negative features, respectively (Table S1). To assess the reproducibility of the metabolome data, Principal component analysis (PCA) was conducted on samples from each treatment. The results indicated that in positive ions, the variability was low and the sample reproducibility was good, whereas the dispersion between different treatments was well-separated, suggesting differences in dehydration response strategies (Figure 1A,B). Further analysis of the expression patterns of all ions showed that the 0% treatment first clustered with the rehydration group (R), followed by clustering with the 30% group (light dehydration) into a major branch, while the 60% (moderate dehydration) and 90% (severe dehydration) groups clustered into another major branch (Figure 1C,D). This suggests that dehydration treatment significantly altered the metabolic activities of the *P. haitanensis*, and 60% dehydration may represent a critical threshold, with the thallus largely recovering to their initial metabolic levels after 2 h of rehydration. To elucidate the tolerance mechanisms of *P. haitanensis*, differentially expressed metabolites were screened based on the criteria of VIP ≥ 1, |Fold Change| ≥ 1.2, and *q*-value < 0.05. The results showed that as the degree of dehydration increased, the number of differential ions gradually increased, while the number of differential ions decreased after rehydration (Figure 1E).

### 2.2. The Adjustments of Osmotic System

**Figure 2:**
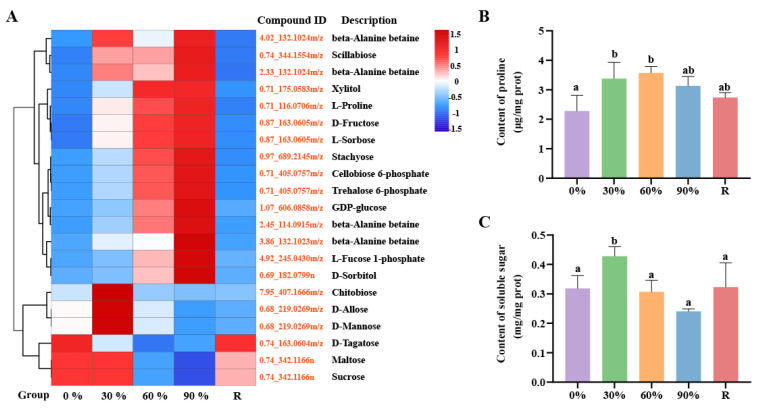
Changes of organic osmolytes content in blades of *P. haitanensis* under dehydration treatment. (**A**) Clustering analysis of organic osmolytes. Compound ID, composed of retention time and *m*/*z*, formatted as ‘RT_*m*/*z*’ (Retention Time_*m*/*z*). For detailed information, refer to Table S2. (**B**) Determination of soluble sugar content. (**C**) Determination of proline content. R represents rehydration for 2 h, and the bar of each column with different small letters means significant difference (*p* < 0.05, Least Significant Difference).

This study found that during the response to dehydration stress, *P. haitanensis* induces the accumulation of sugars including chitobiose, D-mannose, trehalose, and D-allose, as well as sugar alcohols such as xylitol and inositol, and nitrogen-containing and quaternary ammonium compounds represented by proline and beta-Alanine betaine, respectively (Figure 2A). Specifically, during the early stage of dehydration (30%), the primary response to stress is through the accumulation of sugars. As the degree of dehydration intensifies, the accumulation of nitrogen-containing compounds and sugar alcohols begins. Consistent with this observation, measurements of soluble sugar and proline content revealed that compared to normal conditions, the soluble sugar content in the *P. haitanensis* significantly increased under 30% dehydration treatment, while the proline content significantly increased under both 30% and 60% dehydration conditions (Figure 2B,C).

### 2.3. The Activation of Antioxidant Systems

**Figure 3:**
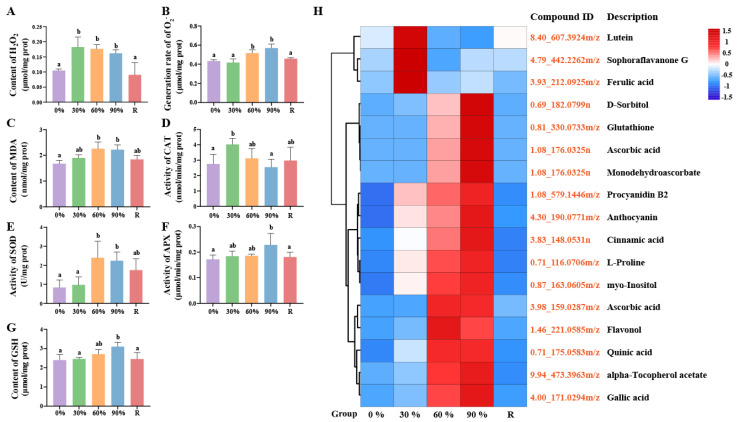
Comparisons of reactive oxygen species (ROS), malondialdehyde (MDA) and antioxidant activities or contents in *P. haitanensis* thalli under different dehydration stress conditions. (**A**) Hydrogen peroxide (H_2_O_2_). (**B**) Superoxide (O_2_^−^). (**C**) MDA content. (**D**) Catalase (CAT). (**E**) Superoxide dismutase (SOD). (**F**) Ascorbate peroxidase (APX). (**G**) Glutathione (GSH). (**H**) Clustering analysis of the differentially expressed metabolites involved in antioxidant system. Compound ID, composed of retention time and *m*/*z*, formatted as ‘RT_*m*/*z*’ (Retention Time_*m*/*z*). For detailed information, refer to Table S3. R represents rehydration for 2 h, and the bar of each column with different small letters means significant difference (*p* < 0.05, Least Significant Difference).

This study found that compared to the control treatment, the content of H_2_O_2_ in the *P. haitanensis* significantly increased after three levels of dehydration treatment (Figure 3A). The generation rate of O_2_^−^ barely changed under 30% dehydration treatment, but it significantly increased once the dehydration rate exceeded 60% (Figure 3B), which could further exacerbate ROS-induced damage to the membrane system, as evidenced by the significant accumulation of malondialdehyde (MDA) content (Figure 3C). However, after rehydration treatment, the contents of H_2_O_2_ and MDA, as well as the generation rate of O_2_^−^, all returned to normal physiological levels. In the face of the massive accumulation of ROS, antioxidant enzymes serve as a crucial defense line, playing an indispensable role in maintaining the balance of ROS in the organism. Therefore, we measured the activity of antioxidant enzymes in the algae. The results indicated that under 30% dehydration treatment, only the content of catalase (CAT) showed a significant increase (Figure 3D). When the dehydration rate exceeded 60%, the activity of superoxide dismutase (SOD) began to increase significantly (Figure 3E). In contrast, ascorbate peroxidase (APX) and GSH only exhibited significant increases under 90% treatment (Figure 3F,G). The activities of these four antioxidant enzymes returned to normal physiological levels after rehydration treatment. In addition to the efficient antioxidant enzyme system, small molecule antioxidants also play a crucial role in ROS scavenging [30]. In the metabolomic analysis, we observed a significant increase in the content of glutathione, as well as various flavonoid antioxidants (sophoraflavanone G, flavonol, anthocyanin, and procyanidin B2), vitamins (ascorbate and tocopherol acetate), phenolic acid compounds (ferulic acid, quinic acid, gallic acid, and cinnamic acid), and amino acids (proline) as small molecule antioxidants, which significantly accumulated in the *P. haitanensis* after dehydration stress (Figure 3H). These substances collectively constitute a robust antioxidant network in the *P. haitanensis*, effectively mitigating the damage caused by ROS.

### 2.4. The Adjustments of Fatty Acids Metabolism

**Figure 4:**
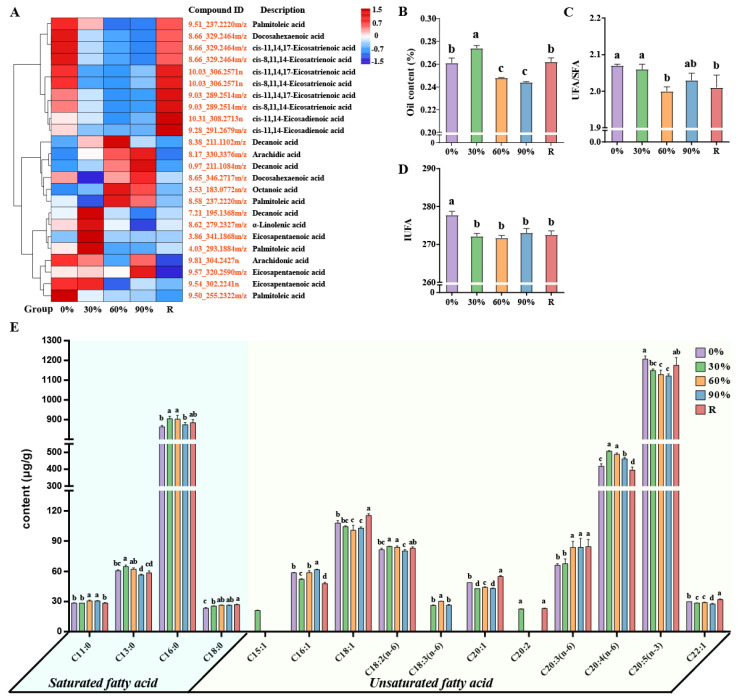
Changes in fatty acids (FAs) and related indexes of *P. haitanensis* under different dehydration stress conditions. (**A**) Cluster analysis of differentially expressed metabolites in fatty acid synthesis. Compound ID, composed of retention time and *m*/*z*, formatted as ‘RT_*m*/*z*’ (Retention Time_*m*/*z*). For detailed information, refer to Table S4. (**B**) Content of total FAs. (**C**) Ratio of unsaturated fatty acids (UFAs) to saturated fatty acids (SFAs). (**D**) Changes of index of unsaturated fatty acid (IUFA) value under different dehydration stress. (**E**) The content of various FAs under different dehydration stress conditions. The bar of each column with different small letters means significant difference (*p* < 0.05, Least Significant Difference).

This study found that *P. haitanensis* can dynamically reorganize their fatty acids (FAs) composition in response to dehydration stress. Specifically, under dehydration stress, *P. haitanensis* inhibit the synthesis of unsaturated fatty acids (UFAs) such as palmitoleic acid (C16:1), cis-11,14,17-eicosatrienoic acid (C20:3 N3), cis-8,11,14-eicosatrienoic acid (C20:3 N6), cis-11,14-eicosadienoic acid (C20:2), and docosahexaenoic acid (C22:6), while promoting the synthesis of saturated fatty acids (SFAs) such as decanoic acid (C10:0), arachidic acid (C20:0), and octanoic acid (C8:0) (Figure 4A). It is noteworthy that, despite the overall trend of inhibited unsaturated fatty acid synthesis, dehydration stress actually stimulates the synthesis of α-linolenic acid (C18:3), arachidonic acid (C20:4), and eicosapentaenoic acid (C20:5) in *P. haitanensis*. In addition, after rehydration treatment, the content of UFAs including C20:2, C20:3 N3, and C20:3 N6 returned to normal levels (Figure 4A). Through further targeted metabolomics analysis, we found that 30% treatment significantly increased the content of FAs in *P. haitanensis* compared with the control treatment. The content of FAs in the thallus decreased significantly under 60% and 90% treatment. It is worth mentioning that after rehydration for two hours, the content of FAs in the thallus returned to normal level (Figure 4B). Compared with the control treatment, the content of UFAs in the thallus decreased significantly under 60% and rehydration treatment (Figure 4C). Meanwhile, the index of unsaturated fatty acid (IUFA) of the thallus also showed a significant downward trend after being subjected to different gradients of dehydration stress, and it remained at a low level two hours after rehydration (Figure 4D). In addition, the SFAs of the thallus are mainly composed of C16:0, and the UFAs are mainly composed of C20:5 (n-3) and C20:4 (n-6). There are also significant differences in the content and proportion of FAs under different treatments (Figure 4E).

### 2.5. The Adjustments of Plant Hormones Metabolism

**Figure 5:**
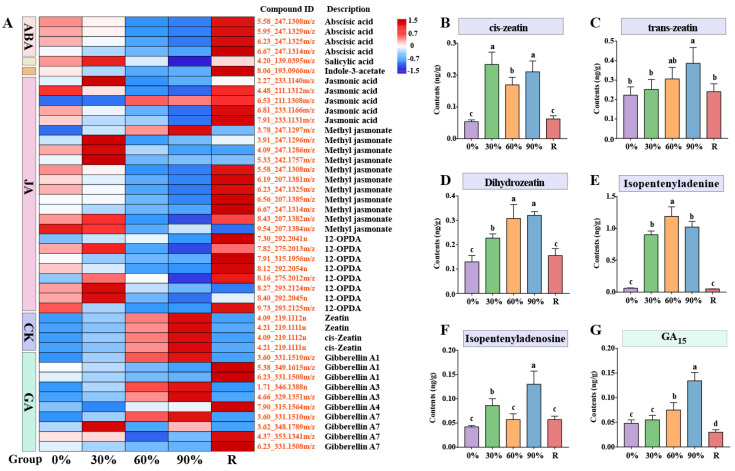
Analysis of plant hormone response strategies of *P. haitanensis* under different dehydration treatment. (**A**) Clustering analysis of plant hormone. Compound ID, composed of retention time and *m*/*z*, formatted as ‘RT_*m*/*z*’ (Retention Time_*m*/*z*). For detailed information, refer to Table S5. B-G Determination of plant hormone content in *P. haitanensis* under different dehydration treatments. (**B**) cis-zeatin. (**C**) trans-zeatin. (**D**) Dihydrozeatin. (**E**) Isopentenyladenine. (**F**) Isopentenyladenosine. (**G**) GA_15_. The bar of each column with different small letters means significant difference (*p* < 0.05, Least Significant Difference).

Through metabolomic analysis, this study identified dynamic changes in the content of various plant hormones in *P. haitanensis* in relation to varying degrees of dehydration. Specifically, the dynamic changes in the contents of salicylic acid (SA) and jasmonic acid (JA) exhibit a distinct biphasic regulation pattern: significant upregulation during the mild dehydration treatment (30%), followed by a significant decrease during severe dehydration treatment (60%); however, following rehydration, the contents of both hormones rebound significantly (Figure 5A). This biphasic regulation pattern indicates that SA and JA not only participate in the early response to mild dehydration stress but may also play crucial regulatory roles in tissue repair and physiological function recovery after rehydration. Indole-3-acetic acid (IAA) and abscisic acid (ABA) showed a significant decline during dehydration but increased markedly upon rehydration, indicating their potential involvement in biological processes associated with the rehydration of *P. haitanensis* (Figure 5A). Conversely, zeatin and various gibberellins (GAs) exhibited an increasing trend in content during dehydration (Figure 5A), hinting at their significant roles in the algae’s resistance to dehydration stress. To further explore the response mechanisms of plant hormones in *P. haitanensis* under dehydration stress, targeted metabolomic analysis was conducted to measure the hormone content in algae subjected to different treatments. Consistent with the metabolomic results, multiple cytokinins (CKs) and CA_15_ showed a significant increasing trend in content during dehydration (Figure 5B–G).

## 3. Discussion

*P. haitanensis*, a seaweed species found in intertidal zones, experiences periodic dehydration and rehydration due to tidal fluctuations. To deeply explore the metabolic response mechanisms of *P. haitanensis* during dehydration and rehydration, this study simulated tidal variations and analyzed the metabolome of *P. haitanensis* under different dehydration gradients. The findings reveal two key strategies employed by *P. haitanensis* in response to desiccation stress: (1) Under dehydration stress, rapidly activate stress response pathways to maintain cellular homeostasis; (2) After rehydration, initiate metabolic reprogramming to promote rapid recovery of key physiological functions such as cell growth and development. The findings aim to elucidate the metabolic mechanisms underlying *P. haitanensis*’s desiccation tolerance, contributing valuable insights for genetic improvement and cultivation strategies.

### 3.1. Osmotic Stress Response Mechanism

Under dehydration stress, plants can regulate osmotic pressure by increasing the levels of four types of organic solutes, including sugars, polyols, nitrogen-containing compounds, and quaternary ammonium compounds. In *P. haitanensis*, the accumulation of these compounds plays a critical role in stabilizing cellular structures and protecting biomacromolecules during dehydration. Specifically, under mild dehydration (30% water loss), *P. haitanensis* primarily accumulates sugars such as chitobiose, D-allose, and D-mannose. As the dehydration stress intensifies to moderate levels (60% water loss), significant increases in the levels of proline, trehalose, xylitol, D-fructose, betaine, and stachyose are observed (Figure 2A–C). Research has revealed that under low-temperature stress conditions, exogenous application of chitobiose can markedly enhance the growth performance of wheat seedlings: it elevates growth parameters such as fresh weight and dry weight of the plants, effectively mitigates the degree of membrane lipid peroxidation, inhibits the reduction in chlorophyll content, and concurrently boosts the accumulation of soluble sugars and the activity of APX [31]. This indicates that chitobiose may augment plant tolerance to abiotic stress by modulating redox balance and osmoprotection mechanisms. Zhao et al., 2020 found that mannose not only functions as a compatible solute to regulate osmotic balance, but also delays leaf senescence by enhancing antioxidant metabolism, suppressing the expression of chlorophyll degradation-related genes, and inducing dehydrin gene expression, thereby significantly improving drought tolerance in white clover [32]. Overall, these osmoprotectants not only help maintain osmotic balance but also contribute to the formation of vitreous structures within cells, which protect against protein denaturation and membrane damage [33,34,35]. Additionally, the accumulation of beta-alanine betaine further enhances cellular osmotic adjustment, reducing membrane damage and preserving enzyme activity [36,37]. These findings are consistent with studies on drought tolerance mechanisms in other species, such as *Selaginella lepidophylla* [33], *Pogonatum inflexum* [38], *Tripogon loliiformis* [39], and *Solanum lycopersicum* [40], where similar osmotic protection strategies have been observed [41,42,43,44].

Concurrently, the adaptive mechanisms employed by *P. haitanensis* during dehydration also contribute to its efficient physiological recovery upon rehydration. Notably, the recovery phase is marked by a rapid restoration of osmotic balance. The levels of soluble sugars are rapidly restored to normal physiological levels, providing essential carbon resources for energy production and macromolecule synthesis (Figure 2C). This metabolic reprogramming supports the rapid recovery of growth-related processes, as evidenced by the upregulation of glycolytic pathway metabolites during rehydration (Figure S1). In summary, *P. haitanensis* employs a dynamic osmotic adjustment strategy, synthesizing osmoprotectants during dehydration and rapidly restoring their levels during rehydration. This dual adaptation strategy not only enhances the alga’s tolerance to desiccation but also supports its efficient recovery, underscoring the critical role of osmotic regulation in its adaptation to the dynamic intertidal environment.

### 3.2. Oxidative Stress Response Mechanism

During the process of dehydration stress, plants reduce their utilization of CO_2_, which leads to disruptions in photosynthesis and subsequently results in the accumulation of ROS [45]. In *P. haitanensis*, the levels of H_2_O_2_ and O_2_^−^ increase significantly during dehydration, particularly under moderate (60%) and severe (90%) water loss (Figure 3A,B). The accumulation of MDA, a marker of lipid peroxidation, further confirms the occurrence of oxidative stress (Figure 3C). However, upon rehydration, the levels of H_2_O_2_, O_2_^−^, and MDA rapidly return to normal physiological levels, indicating the alga’s ability to efficiently scavenge ROS and repair oxidative damage. To mitigate ROS-induced damage, *P. haitanensis* activates a robust antioxidant system. Under mild dehydration (30%), CAT activity increases significantly, while SOD and APX activities rise under moderate and severe dehydration (Figure 3D–F). GSH levels also increase under severe dehydration, further enhancing the antioxidant capacity (Figure 3G). These findings are consistent with studies on other intertidal algae, such as *Gracilaria corticata* [46] and *Porphyra columbina* [47], where similar antioxidant responses have been observed.

In addition to enzymatic antioxidants, small molecule antioxidants play a crucial role in ROS scavenging. During dehydration, *P. haitanensis* accumulates a variety of non-enzymatic antioxidants, including ascorbic acid (vitamin C), flavonoids, and tocopherols (vitamin E), which collectively form a comprehensive antioxidant network (Figure 3H). Ascorbic acid and GSH act as hydrophilic redox buffers, while tocopherols function as liposoluble antioxidants, protecting membrane lipids from oxidative damage [30,48]. Flavonoids, such as anthocyanins and procyanidins, exhibit strong antioxidant activity, directly scavenging free radicals and regenerating other oxidized antioxidants [49]. The synergistic interaction between enzymatic and non-enzymatic antioxidants ensures efficient ROS clearance and minimizes oxidative damage, highlighting the complexity and efficiency of *P. haitanensis*’ antioxidant defense system. In summary, the synergistic action of enzymatic and non-enzymatic antioxidants ensures efficient ROS scavenging and minimizes oxidative damage, demonstrating the complexity and efficiency of its antioxidant defense system. This dynamic response highlights the critical role of ROS scavenging in its adaptation to the fluctuating intertidal environment and provides valuable insights into the metabolic mechanisms underlying its stress tolerance.

### 3.3. Fatty Acid Metabolism Response Mechanism

Lipids exist throughout the entire life cycle of plants and are not only the main components of biological membranes, but also play important roles in energy conversion, signal transduction, and other aspects. The plant cell membrane system is highly sensitive to external stress, and dehydration can disrupt membrane structure and function. FAs, as the main components of the plasma membrane, play a critical role in maintaining membrane integrity under stress conditions [50]. In *P. haitanensis*, the content and composition of FAs dynamically change in response to dehydration. Under mild dehydration (30%), the alga increases FA synthesis to maintain membrane integrity. However, as dehydration intensifies (60% and 90% water loss), FA content decreases significantly, accompanied by a rise in MDA levels, a marker of lipid peroxidation, indicating membrane damage caused by dehydration stress (Figure 3C and Figure 4A,B). Remarkably, upon rehydration, FA levels rapidly return to normal, demonstrating the *P. haitanensis*’s strong membrane repair capacity (Figure 4B). Dehydration also alters the balance between SFAs and UFAs. While SFAs content remains stable under mild dehydration, UFAs levels and the UFAs/SFAs ratio decrease significantly under moderate and severe dehydration, as well as after rehydration (Figure 4C). This shift toward higher SFAs content reduces membrane unsaturation, minimizing lipid peroxidation and membrane damage caused by ROS [51,52]. This adaptation helps maintain membrane integrity under stress conditions. Additionally, increasing the saturation of membrane lipids reduces cell membrane permeability and mitigates damage caused by osmotic stress [53].

The IUFA value reflects membrane lipid fluidity, with higher IUFA values indicating greater unsaturation and fluidity [54]. In this study, the IUFA value of *P. haitanensis* showed a significant downward trend after dehydration stress, particularly under mild dehydration (30% water loss) (Figure 4D). Notably, at this stage, the levels of MDA remained normal, suggesting that the alga mitigates membrane damage by reducing FA unsaturation. This adaptation helps maintain membrane integrity and reduces permeability, thereby preserving cellular turgor pressure and supporting growth under stress conditions. Similar findings have been reported in *Artemisia sphaerocephala*, where increased SFA content and reduced UFA levels under drought stress minimized lipid peroxidation and membrane damage [55].

Interestingly, despite the overall reduction in UFAs, the levels of C18:3 and C20:4 increased under dehydration stress (Figure 4A,E). C18:3 not only enhances membrane fluidity but also functions as a signaling molecule during stress responses [56,57]. Studies have shown that high levels of C18:3 help maintain photosynthesis and reduce membrane damage under drought stress [58,59,60]. Similarly, C20:4 plays a critical role in stabilizing chloroplast membranes and ensuring photosynthesis under stress conditions, as observed in *Lobosphaera incisa* [61] and *Phoenix dactylifera* [62]. These findings highlight the dual roles of polyunsaturated fatty acids in membrane protection and stress signaling. The increase in C20:4 content, coupled with the decrease in its precursors (C20:2 and C20:3), highlights the alga’s proactive metabolic adaptation to stress (Figure 4A). This metabolic reprogramming reflects a strategic shift in lipid metabolism, with *P. haitanensis* prioritizing the synthesis of C20:4—a key polyunsaturated fatty acid for membrane stability and stress signaling [63]—over its precursors. Such a shift not only enhances membrane integrity under dehydration stress but also supports the alga’s ability to rapidly recover upon rehydration. In summary, the dynamic changes in FA composition, including the selective accumulation of C18:3 and C20:4, underscore the importance of lipid metabolism in *P. haitanensis*’ stress response. The rapid recovery of FA levels after rehydration highlights the alga’s metabolic flexibility, enabling it to balance growth and stress tolerance in the fluctuating intertidal

### 3.4. Plant Hormones Metabolism Response Mechanism

Plants exhibit remarkable adaptability to variable environmental factors through the delicate regulation of their hormones. For instance, JA effectively mitigates membrane damage by increasing the content of osmolytes such as proline in plants, thereby enhancing their stress resistance [64]. Abouelsaad et al., 2018 found that JA can also maintain the dynamic balance of reactive oxygen species by activating enzymatic and non-enzymatic antioxidant systems, thereby significantly enhancing the salt tolerance of tomatoes [65]. Similarly, SA can enhance plant tolerance to abiotic stress by regulating photosynthesis, metabolite accumulation, redox homeostasis, and gene regulation [66]. Liu et al., 2024 found in their study of sunflowers that SA can not only alleviate light damage caused by salt stress by enhancing the efficiency of photosystem II, but also effectively reduce ROS accumulation by activating antioxidant systems (such as SOD, CAT, POD activity), thereby protecting the cell structure of sunflowers and improving their physiological status [67].

This study further delves into the dynamic changes in plant hormone content in *P. haitanensis* when facing dehydration stress. Specifically, under mild dehydration stress (30%), the content of SA and JA in the cells of *P. haitanensis* significantly increases (Figure 5A), indicating that these two plant hormones play vital roles in protecting the biological macromolecules of the *P. haitanensis*, reducing intracellular ROS content, and alleviating membrane lipid peroxidation, thereby helping *P. haitanensis* maintain its normal physiological functions under mild dehydration conditions. However, when dehydration stress reaches a moderate level (60%), the hormonal response mechanism of *P. haitanensis* seems to shift, with the thallus responding to stress by upregulating the content of zeatin and various GAs (Figure 5A–F). Further targeted metabolomic analysis demonstrated that GA_15_ is the predominant GA form accumulated in *P. haitanensis* under dehydration stress (Figure 5G). As a key intermediate in the GAs biosynthetic pathway, GA_15_ dynamically balances active and inactive GA pools by regulating GA20-oxidase activity [68,69]. This metabolic “buffering” mechanism stabilizes GA signaling flux under extreme desiccation, ensuring sustained transmission of basal growth signals while avoiding overactivation of bioactive GAs [70]. Similar phenomena have been observed in *Arabidopsis* [71] and tomato [72], where organisms reduce bioactive GA levels in response to drought stress. On the other hand, as a key cytokinin, zeatin plays an important role in plant stress resistance, and studies have shown that exogenous cytokinin application significantly improves plant drought tolerance [73]. Therefore, we propose that *P. haitanensis* enhances drought resistance under severe dehydration stress through coordinated accumulation of zeatin and GA_15_, which may serve as an effective strategy for adapting to extreme environments. The preferential accumulation of GA_15_ likely reflects adaptive evolution in intertidal organisms to balance “growth and stress resistance” under periodic dehydration pressure. These findings suggest that *P. haitanensis* employs a multi-layered hormonal strategy to cope with varying degrees of dehydration stress.

Upon rehydration, the hormonal profile of *P. haitanensis* exhibits a new regulatory pattern, characterized by significant accumulation of JA, IAA, and ABA. The increase in JA levels likely facilitates the repair of stress-induced damage by enhancing antioxidant capacity and stabilizing membrane integrity. IAA, as a key auxin [74], is markedly upregulated during rehydration, potentially driving rapid thallus recovery by stimulating cell expansion and tissue repair. Unlike the pattern in most terrestrial plants where ABA primarily accumulates during dehydration stress to trigger stomatal closure and drought avoidance [75], *P. haitanensis* exhibits a unique ABA accumulation pattern during the rehydration process. We propose that this temporal shift in ABA dynamics might reflects a novel evolutionary adaptation to its intertidal habitat, where predictable cycles of desiccation and rehydration necessitate prioritization of rapid recovery over immediate water conservation. This is supported by Zhang et al., 2022, who demonstrated that ABA pretreatment enhances rehydration efficiency in *P. haitanensis*, suggesting a functional shift toward recovery optimization [76]. Furthermore, ABA accumulation may serve to activate additional physiological mechanisms, including gene expression and metabolic pathway adjustments, to support the alga’s rapid recovery from stress [77]. Additionally, ABA accumulation after rehydration may also function as a post-stress “memory” mechanism [78], which is of critical importance for the alga’s adaptation to the periodically changing intertidal environment. Collectively, the synergistic actions of JA, IAA, and ABA ensure a flexible transition from stress response to growth and development, highlighting the alga’s efficient adaptation strategy to intertidal environments.

## 4. Materials and Methods

### 4.1. Materials and Desiccation Treatment

*Pyropia haitanensis* strain Z-61 used in this research was from the Laboratory of Germplasm Improvements and Applications of *Pyropia* in Jimei University, Fujian, China [79]. Blades were cultured in a growth chamber with Provasoli’s enriched seawater under 50 μmol photons m^−2^ s^−1^ at 21 °C and a 12:12 light:dark photoperiod. PES was changed every three days. Blades of Z-61 grow to 15 ± 2 cm were randomly selected for stress treatment. According to the water loss, four different desiccation levels were set, including 0% water loss (control), 30% water loss (mild desiccation), 60% water loss (moderate desiccation), and 90% water loss (severe desiccation). Rehydration treatment was performed by submerging the blades for 2 h after the water loss reached 90%. Water loss rate was calculated according to previous study [23].

### 4.2. Physiological Measurements

#### 4.2.1. Soluble Protein Content Determination

The soluble protein content was determined using the BCA method. Samples were ground in liquid nitrogen, and the analysis was performed as follows: Reagent A contained 1% BCA, 2% Na_2_CO_3_, 0.16% sodium tartrate, 0.4% NaOH, and 0.95% NaHCO_3_ (pH 11.5), while Reagent B contained 4% CuSO_4_. The working solution was prepared by mixing 100 mL of Reagent A with 2 mL of Reagent B. A standard curve was established using bovine serum albumin (BSA) at 1.5 mg/mL. For each measurement, 0.1 mL of diluted sample was mixed with 1 mL of working solution, incubated at 37 °C for 30 min, and the absorbance was measured at 562 nm. Protein concentrations were calculated using the standard curve. All subsequent biochemical measurements were normalized to the protein content and expressed per milligram protein (mg prot). Three biological replicates were analyzed for each treatment.

#### 4.2.2. Determination of Reactive Oxygen Species and Malondialdehyde Content

H_2_O_2_ content was determined by homogenizing 0.1 g samples in 1 mL of 5% TCA on ice, followed by centrifugation at 8000× *g* for 10 min at 4 °C. The supernatant was analyzed using a commercial kit (H_2_O_2_-2-Y, Cominbio, Suzhou, China) based on the reaction of H_2_O_2_ with titanium sulfate to form a yellow peroxidized titanium complex, which exhibits a characteristic absorption peak at 415 nm. Regarding O_2_^−^, due to its extremely short lifetime, direct determination of its content is not feasible. Therefore, the generation rate of O_2_^−^ was measured instead. The procedure was similar to that for H_2_O_2_ content determination, except that centrifugation was performed at 10,000× *g*, and a specific commercial kit (SA-2-G, Cominbio, Suzhou, China) was used for analysis. The assay relies on the reaction of O_2_^−^ with hydroxylamine hydrochloride to generate NO_2_^−^, which subsequently reacts with sulfanilic acid and α-naphthylamine to form a red azo compound. The absorbance at 530 nm was measured to calculate O_2_^−^ levels in the samples. For MDA determination, samples were homogenized in 10% TCA and centrifuged. The supernatant was mixed with 0.5% TBA and 1% phosphoric acid (1:6:2 ratio), heated at 100 °C for 45 min, then extracted with n-butanol (2500× *g*, 5 min). Absorbance at 532 nm was measured and MDA content (nmol/mg protein) was calculated using a standard curve [80].

#### 4.2.3. Determination of Antioxidant Enzyme Activities

Fresh samples (0.1 g) were homogenized in 1 mL of the corresponding extraction buffer on ice, followed by centrifugation at 8000× *g* for 10 min at 4 °C. The resulting supernatant was collected and analyzed for SOD, APX, and CAT activities, as well as GSH content, using commercial kits (Cominbio, Suzhou, China) [81,82].

SOD activity (SOD-2-Y kit) determination principle: Xanthine/xanthine oxidase-generated O_2_^−^ reduces nitroblue tetrazolium (NBT) to blue formazan (560 nm absorbance), with SOD activity inversely proportional to formazan formation.

APX activity (APX-2-W kit) determination principle: APX catalyzes H_2_O_2_ reduction by oxidizing ascorbic acid, quantified via ascorbic acid oxidation rate.

CAT activity (CAT-2-Y kit) determination principle: H_2_O_2_ exhibits a characteristic absorption peak at 240 nm. CAT decomposes H_2_O_2_, causing the absorbance of the reaction solution at 240 nm to decrease over time. The activity of CAT can be calculated based on the rate of absorbance change.

GSH content (GSH-2-W kit) determination principle: GSH reacts with 5,5’-dithiobis-2-nitrobenzoic acid (DTNB) to form a yellow complex (412 nm absorbance proportional to concentration).

#### 4.2.4. Determination of Osmotic Regulation Substances

Soluble sugar content was determined by homogenizing 0.1 g samples in 1 mL distilled water. The homogenates were heated to 95 °C for 10 min, then centrifuged at 8000× *g* for 10 min at 25 °C. The resulting supernatant was analyzed using a commercial kit (KT-2-Y, Cominbio, Suzhou, China). For proline determination, 0.1 g samples were homogenized in 0.9 mL extraction buffer on ice, followed by centrifugation at 3500× *g* for 10 min at 4 °C. The supernatant was analyzed according to the manufacturer’s protocol (PRO-2-Y, Cominbio, Suzhou, China).

### 4.3. Metabolite Extraction and Measurement

A total of 25 mg of the sample was placed into an EP tube. A total of 800 μL of pre-cooled methanol/water (1:1) tempering solution and two small steel balls were added to each EP tube. The sample was placed in the tissue Lyser and the parameter was set to 50 HZ for 4 min. After grinding, the steel ball was removed and placed in the EP tube in a −20 °C refrigerator for 2 h. It was centrifuged at 30,000× *g* for 20 min at 4 °C, and 550 μL of each sample was placed in a new EP tube. Mix 35 μL of each sample into QC samples, dispense all samples into 96-well plates at 60 μL/well, and sequence using LC-MS referenced on Dunn et al., 2011 [83]. Six biological replicates were analyzed for each treatment.

### 4.4. Liquid Phase Parameters

Chromatographic separation was performed using an ACQUITY UPLC HSS T3 column (100 mm × 2.1 mm, 1.8 μm, Waters, Milford, MA, USA), with the column temperature maintained at 50 °C and a flow rate of 0.4 mL/min. Mobile phase A consisted of water with 0.1% formic acid, while mobile phase B was methanol with 0.1% formic acid. The metabolites were eluted using the following gradient: 0–2 min, 100% mobile phase A; 2–11 min, 0–100% mobile phase B; 11–13 min, 100% mobile phase B; and 13–15 min, 0–100% mobile phase A. The injection volume for each sample was 10 μL.

### 4.5. Mass Spectrometry Parameters

For the small molecules eluted from the chromatographic column, high-resolution tandem mass spectrometry using an Xevo G2-XS QTOF (Waters, Milford, MA, USA) was employed to collect data in both positive and negative ion modes. In positive ion mode, the capillary voltage and cone voltage were set at 3 kV and 40 V, respectively. For negative ion mode, the capillary voltage and cone voltage were adjusted to 2 kV and 40 V, respectively. Data acquisition in centroid mode was performed using MSE (Multiple Stage Energy) mode, with a primary scan range of 50–1200 Da and a scan time of 0.2 s. All parent ions were fragmented with an energy ranging from 20 to 40 eV to collect all fragment information, with a scan time of 0.2 s. During data acquisition, real-time mass calibration was conducted every 3 s for the LE signal. Additionally, a pooled quality control sample was acquired every 10 samples to assess the stability of the instrument status during the sample acquisition process.

### 4.6. Peak Extraction and Identification

The metabolomics data processing was performed using Progenesis QI (version 2.2) through an integrated workflow that included peak alignment (automatically selecting the optimal QC sample as reference), peak extraction (using automatic_default parameters), data normalization (normalize to all compounds), and compound identification (matched against KEGG database with 10 ppm mass tolerance). In positive ion mode, detected adducts included [M+H]^+^, [M+NH_4_]^+^, [M+K]^+^, [M+Na]^+^, and [M+H−H_2_O]^+^, while negative mode detected [M−H]^−^ and [M+Cl]^−^. Subsequent data preprocessing in metaX (version 2.0.0) software implemented missing value imputation using K-nearest neighbors (KNN) algorithm [84], removal of low-quality ions (absent in >50% QC samples or >80% experimental samples), and quality filtering (excluding ions with RSD >30% across QCs) (Figure S2), ultimately yielding 7975 positive and 5401 negative ions for downstream analysis to ensure data reproducibility and reliability.

### 4.7. Screening of Differential Ions

Difference ions were screened using the VIP values of the first two principal components from a multivariate PLS-DA model, in combination with univariate analysis of fold change and *q*-value. The screening criteria were (1) VIP > 1; (2) fold change > 1.2 or ≤0.833; (3) *q*-value < 0.05. The intersection of these three conditions was taken to identify common ions, which were considered as differential ions.

### 4.8. Determination of Fatty Acids Contents

Approximately 200 mg of freeze-dried *P. haitanensis* sample was homogenized and mixed with 50 μL of C17:0 methyl ester internal standard solution (5 mg/mL in petroleum ether, 90–120 °C), 2 mL of 5% (*v*/*v*) sulfuric acid-methanol solution, and 300 μL toluene. The mixture was transferred to a headspace vial, sealed with a PTFE-lined aluminum cap, gently vortexed, and heated at 95 °C for 1.5 h for methylation. After cooling to room temperature, 2 mL of 0.9% (*w*/*v*) NaCl solution was added, followed by extraction with 1 mL n-hexane and centrifugation to collect the supernatant for GC analysis.

The supernatant was analyzed using an Agilent 7890A (Agilent Technologies, Santa Clara, CA, USA) equipped with a DB-FastFAME capillary column (30 m × 0.25 mm × 0.25 μm) and a flame ionization detector (FID) maintained at 260 °C. The injection port temperature was set at 250 °C with a split ratio of 20:1. The oven temperature program consisted of initial temperature at 80 °C (hold 0.5 min), ramp at 40 °C/min to 165 °C (hold 1 min), followed by a 4 °C/min increase to 230 °C (hold 4 min). Fatty acids were identified by retention time matching with authentic standards and quantified using the formula: Lipid content = (total peak area S1/internal standard peak area S2) × (internal standard amount N/sample weight M). Four biological replicates were analyzed for each treatment [85].

### 4.9. Determination of Plant Hormone Content

Fresh *P. haitanensis* materials were harvested, weighed, frozen in liquid nitrogen, and stored at −80 °C. For extraction, 50 mg of plant material was powdered under liquid nitrogen and extracted with 1 mL of methanol/water/formic acid (15:4:1, *v*/*v*/*v*). Extracts were dried under nitrogen, reconstituted in 100 μL of 80% methanol, and filtered through a 0.22 μm membrane for LC-MS analysis.

Sample extracts were analyzed using an LC-ESI-MS/MS system (UHPLC ExionLC™ AD and Applied Biosystems 6500 Triple Quadrupole, Sciex, ON, Canada) [86]. The HPLC column was Waters ACQUITY UPLC HSS T3 C18 (Waters, Milford, MA, USA) (100 mm × 2.1 mm i.d., 1.8 μm). The solvent system consisted of water with 0.04% acetic acid (A) and acetonitrile with 0.04% acetic acid (B). The gradient program was 5% B (0–1 min), increased to 95% B (1–8 min), held at 95% B (8–9 min), and returned to 5% B (9.1–12 min). The flow rate was 0.35 mL/min, temperature was 40 °C, and injection volume was 2 μL.

The AB 6500+ QTRAP^®^ LC-MS/MS System (SCIEX, Framingham, MA, USA), equipped with an ESI Turbo Ion Spray interface and controlled by Analyst 1.6 software, operated in both positive and negative ion modes. ESI source parameters included turbo spray source, temperature of 550 °C, ion spray voltage of 5500 V (positive) and −4500 V (negative), and curtain gas set at 35.0 psi. DP and CE for individual MRM transitions were optimized, and specific MRM transitions were monitored for each period based on the eluted plant hormones. The detailed mass spectrometry detection method parameters are provided in Table S6. Four biological replicates were analyzed for each treatment.

### 4.10. Data Processing and Statistical Analysis

The significance of any differences between the treatment and control values was determined with a one-way ANOVA and the Least Significant Difference post hoc test in SPSS 13.0 (SPSS Inc., Chicago, IL, USA) (*p* < 0.05).

## 5. Conclusions

**Figure 6:**
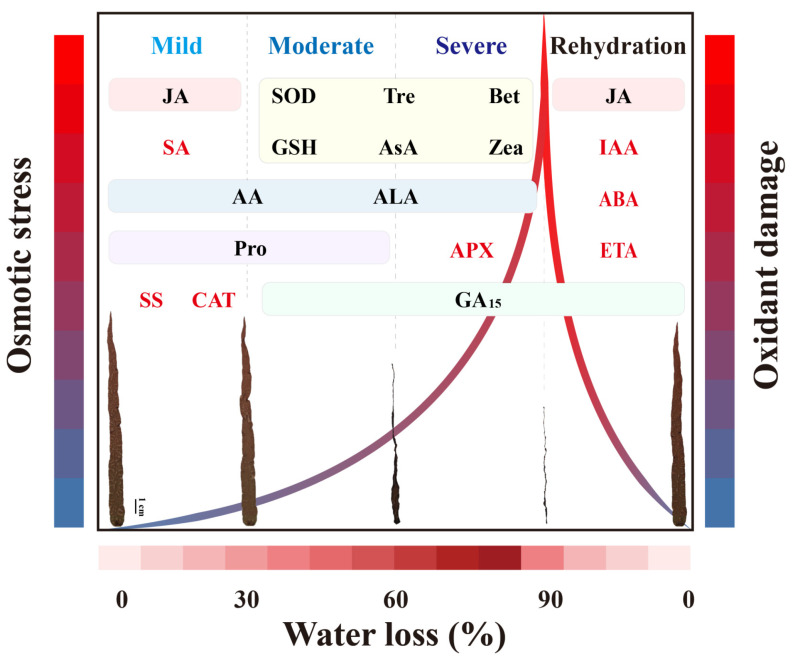
Model explaining the possible mechanisms underlying the desiccation tolerance of *P. haitanensis* depending on the metabolomics analysis. As the degree of water loss intensifies, the osmotic stress and oxidative damage experienced by the *P. haitanensis* also progressively increase, returning to normal physiological status after rehydration. Specifically, during mild dehydration (30%), the thalli surface began to darken in color and lose gloss; at moderate dehydration (60%), the blades showed significant contraction with deepened wrinkles; and at severe dehydration (90%), the thalli completely shrink and lose their toughness. Among these, the substances highlighted in red font represent unique response substances specific to that particular degree of water loss, while the substances enclosed in boxes of different colors signify common response substances across multiple different levels of water stress. SA, salicylic acid; SS, soluble sugar; JA, jasmonic acid; Pro, proline; CAT, catalase; Tre, trehalose; AsA, ascorbic acid; GSH, glutathione; GA_15_, gibberellin GA_15_; Zea, zeatin; APX, ascorbate peroxidase; Bet, betaine; IAA, Indole-3-acetic acid; ABA, abscisic acid; AA, arachidonic acid; ALA, α-Linolenic acid; ETA, Eicosatrienoic acid.

This study reveals the multifaceted metabolic strategies of *P. haitanensis* to cope with desiccation stress. Under dehydration, the *P. haitanensis* accumulates sugars, proline, and betaines to maintain osmotic balance and stabilizes cellular structures. It also activates a robust antioxidant system, including enzymes (SOD, APX, CAT) and non-enzymatic compounds (ascorbic acid, GSH, flavonoids), to counteract ROS-induced oxidative damage. Additionally, *P. haitanensis* modifies its membrane lipid composition by reducing unsaturation and increasing SFAs, while enhancing levels of α-linolenic acid and arachidonic acid for membrane fluidity and stress signaling. Hormonal regulation plays a dynamic role: SA and JA accumulate under mild stress to protect cells, while CKs and GA_15_ dominate under severe dehydration to enhance stress tolerance. After rehydration, the hormone balance shifts again, with JA, IAA, and ABA accumulating to stimulate growth and facilitate tissue repair (Figure 6). These adaptive mechanisms highlight *P. haitanensis*’ resilience to fluctuating intertidal conditions, offering valuable insights for improving its cultivation and stress tolerance.

## Data Availability

The data presented in this study are available on request from the corresponding author.

## Supplementary Materials

The following supporting information can be downloaded at: https://www.mdpi.com/article/10.3390/md23050203/s1. Figure S1: Clustering analysis of the differentially expressed metabolites involved in glycolytic pathway; Figure S2: Principal component analysis of QC samples. (**A**) Positive ions mode. (**B**) Negative ions mode; Table S1: Summary of filtered positive and negative ion features based on relative standard deviation (RSD) in quality control (QC) samples; Table S2: Signal intensity and annotation information of differentially expressed organic osmolytes in *P. haitanensis* under different dehydration treatments; Table S3: Signal intensity and annotation information of differentially expressed antioxidant system in *P. haitanensis* under different dehydration treatments; Table S4: Signal intensity and annotation information of differentially expressed fatty acids in *P. haitanensis* under different dehydration treatments; Table S5: Signal intensity and annotation information of differentially expressed plant hormone in *P. haitanensis* under different dehydration treatments; Table S6: Mass spectrometry detection method parameters for plant hormones in *P. haitanensis*.

## Abbreviations

The following abbreviations are used in this manuscript:

ABA	abscisic acid
APX	ascorbate peroxidase
C10:0	decanoic acid
C16:1	Palmitoleic acid
C18:3	α-linolenic acid
C20:0	arachidic acid
C20:2	cis-11,14-Eicosadienoic acid
C20:3 N3	cis-11,14,17-Eicosatrienoic acid
C20:3 N6	cis-8,11,14-Eicosatrienoic acid
C20:4	arachidonic acid
C20:5	eicosapentaenoic acid
C22:6	docosahexaenoic acid
C8:0	octanoic acid
CAT	catalase
CKs	cytokinins
FA	fatty acid
GAs	gibberellins
GSH	glutathione
IAA	Indole-3-acetic acid
IUFA	index of unsaturated fatty acid
JA	jasmonic acid
MDA	malondialdehyde
ROS	reactive oxygen species
SA	salicylic acid
SFA	saturated fatty acids
SOD	superoxide dismutase
UFA	unsaturated fatty acids

## References

[1] Dittami S.M., Scornet D., Petit J.-L., Ségurens B., Da Silva C., Corre E., Dondrup M., Glatting K.-H., König R., Sterck L. (2009). Global expression analysis of the brown alga *Ectocarpus siliculosus* (Phaeophyceae) reveals large-scale reprogramming of the transcriptome in response to abiotic stress. Genome Biol..

[2] Blouin N.A., Brodie J.A., Grossman A.C., Xu P., Brawley S.H. (2011). *Porphyra*: A marine crop shaped by stress. Trends Plant Sci..

[3] Brawley S.H., Blouin N.A., Ficko-Blean E., Wheeler G.L., Lohr M., Goodson H.V., Jenkins J.W., Blaby-Haas C.E., Helliwell K.E., Chan C.X. (2017). Insights into the red algae and eukaryotic evolution from the genome of *Porphyra umbilicalis* (Bangiophyceae, Rhodophyta). Proc. Natl. Acad. Sci. USA.

[4] Huan L., Xie X., Zheng Z., Sun F., Wu S., Li M., Gao S., Gu W., Wang G. (2014). Positive correlation between PSI response and oxidative pentose phosphate pathway activity during salt stress in an intertidal macroalga. Plant Cell Physiol..

[5] Chen H., Chu J.S.-C., Chen J., Luo Q., Wang H., Lu R., Zhu Z., Yuan G., Yi X., Mao Y. (2022). Insights into the ancient adaptation to intertidal environments by red algae based on a genomic and multiomics investigation of *Neoporphyra haitanensis*. Mol. Biol. Evol..

[6] Yoon H.S., Hackett J.D., Ciniglia C., Pinto G., Bhattacharya D. (2004). A molecular timeline for the origin of photosynthetic eukaryotes. Mol. Biol. Evol..

[7] Wen J., Wang W., Xu K., Ji D., Xu Y., Chen C., Xie C. (2020). Comparative Analysis of Proteins Involved in Energy Metabolism and Protein Processing in *Pyropia haitanensis* at Different Salinity Levels. Front. Mar. Sci..

[8] Wang W., Ge Q., Wen J., Zhang H., Guo Y., Li Z., Xu Y., Ji D., Chen C., Guo L. (2024). Horizontal gene transfer and symbiotic microorganisms regulate the adaptive evolution of intertidal algae, *Porphyra sense lato*. Commun. Biol..

[9] Wang W., Chen T., Xu Y., Xu K., Ji D., Chen C., Xie C. (2020). Investigating the mechanisms underlying the hyposaline tolerance of intertidal seaweed, *Pyropia haitanensis*. Algal Res..

[10] Park J.-S., Jeong Y.-R., Chun B.-S. (2019). Physiological activities and bioactive compound from laver (*Pyropia yezoensis*) hydrolysates by using subcritical water hydrolysis. J. Supercrit. Fluids.

[11] FAO (2025). Fishery and Aquaculture Statistics—Yearbook.

[12] Zhang Z., Wang X., Pan Y., Wang G., Mao G. (2020). The degraded polysaccharide from *Pyropia haitanensis* represses amyloid beta peptide-induced neurotoxicity and memory in vivo. Int. J. Biol. Macromol..

[13] Yang Y., Lin H., Fu X. (2024). Fermentation of *Pyropia* spp. seaweed: A comprehensive review on processing conditions, biological activities and potential applications in the food industry. Crit. Rev. Food Sci. Nutr..

[14] Liu P., Luo Q., Zhu S., Chen J., Yang R., Niu T., Wang T., Zhang P., Chen H. (2025). The extensive cultivation of *Pyropia haitanensis* along the coastal areas influences the ecological dynamics of nearby intertidal mudflat pond water. Front. Mar. Sci..

[15] Gao S., Wang G. (2012). The enhancement of cyclic electron flow around photosystem I improves the recovery of severely desiccated *Porphyra yezoensis* (Bangiales, Rhodophyta). J. Exp. Bot..

[16] Behera D.P., Ingle K.N., Mathew D.E., Dhimmar A., Sahastrabudhe H., Sahu S.K., Krishnan M.G., Shinde P.B., Ganesan M., Mantri V.A. (2022). Epiphytism, diseases and grazing in seaweed aquaculture: A comprehensive review. Rev. Aquac..

[17] Li X.-l., Wang W.-j., Liu F.-l., Liang Z.-r., Sun X.-t., Yao H.-q., Wang F.-j. (2018). Periodical drying or no drying during aquaculture affects the desiccation tolerance of a sublittoral *Pyropia yezoensis* strain. J. Appl. Phycol..

[18] Wang L., Mao Y., Kong F., Cao M., Sun P. (2015). Genome-wide expression profiles of *Pyropia haitanensis* in response to osmotic stress by using deep sequencing technology. BMC Genom..

[19] Im S., Lee H.-N., Jung H.S., Yang S., Park E.-J., Hwang M.S., Jeong W.-J., Choi D.-W. (2017). Transcriptome-based identification of the desiccation response genes in marine red algae *Pyropia tenera* (Rhodophyta) and enhancement of abiotic stress tolerance by PtDRG2 in *Chlamydomonas*. Mar. Biotechnol..

[20] Contreras-Porcia L., López-Cristoffanini C., Meynard A., Kumar M. (2017). Tolerance pathways to desiccation stress in seaweeds. Syst. Biol. Mar. Ecosyst..

[21] Yin J., Sun Y., Miao X., Qu J., Zhang K., Qing Han X., Li Y., Sun J., Kong F. (2025). Dynamic changes and transcriptome analyses reveal the microfilament skeleton response to water stress in thalli of *Neopyropia yezoensis* inhabiting the intertidal zone. Plant Stress.

[22] Xu K., Xu Y., Ji D., Xie J., Chen C., Xie C. (2016). Proteomic analysis of the economic seaweed *Pyropia haitanensis* in response to desiccation. Algal Res..

[23] Shi J., Wang W., Lin Y., Xu K., Xu Y., Ji D., Chen C., Xie C. (2019). Insight into transketolase of *Pyropia haitanensis* under desiccation stress based on integrative analysis of omics and transformation. BMC Plant Biol..

[24] Saito K., Matsuda F. (2010). Metabolomics for functional genomics, systems biology, and biotechnology. Annu. Rev. Plant Biol..

[25] Hama J.R., Hooshmand K., Laursen B.B., Vestergård M., Fomsgaard I.S. (2022). Clover root uptake of cereal benzoxazinoids (BXs) caused accumulation of BXs and BX transformation products concurrently with substantial increments in clover flavonoids and abscisic acid. J. Agric. Food Chem..

[26] Gupta V., Thakur R.S., Baghel R.S., Reddy C., Jha B. (2014). Seaweed metabolomics: A new facet of functional genomics. Adv. Bot. Res..

[27] Ye Y., Yang R., Lou Y., Chen J., Yan X., Tang H. (2014). Effects of food processing on the nutrient composition of *Pyropia yezoensis* products revealed by NMR-based metabolomic analysis. J. Food Nutr. Res..

[28] Liu Y., Qian H., Zhu S., Niu T., Luo Q., Chen J., Yang R., Zhang P., Wang T., Chen H. (2025). Metabolome analysis reveals the involvement of oxylipins in regulating the maturation of conchosporangia in *Pyropia haitanensis*. Algal Res..

[29] Jian Q., Zhu X., Chen J., Zhu Z., Yang R., Luo Q., Chen H., Yan X. (2017). Analysis of global metabolome by gas chromatography-mass spectrometry of *Pyropia haitanensis* stimulated with 1-octen-3-ol. J. Appl. Phycol..

[30] Gill S.S., Tuteja N. (2010). Reactive oxygen species and antioxidant machinery in abiotic stress tolerance in crop plants. Plant Physiol. Biochem..

[31] Zou P., Tian X., Dong B., Zhang C. (2017). Size effects of chitooligomers with certain degrees of polymerization on the chilling tolerance of wheat seedlings. Carbohydr. Polym..

[32] Zhao S., Zeng W., Li Z., Peng Y. (2020). Mannose regulates water balance, leaf senescence, and genes related to stress tolerance in white clover under osmotic stress. Biol. Plant..

[33] Yobi A., Wone B.W., Xu W., Alexander D.C., Guo L., Ryals J.A., Oliver M.J., Cushman J.C. (2013). Metabolomic profiling in *Selaginella lepidophylla* at various hydration states provides new insights into the mechanistic basis of desiccation tolerance. Mol. Plant.

[34] Oliver M.J., Farrant J.M., Hilhorst H.W., Mundree S., Williams B., Bewley J.D. (2020). Desiccation tolerance: Avoiding cellular damage during drying and rehydration. Annu. Rev. Plant Biol..

[35] Bray E.A. (1997). Plant responses to water deficit. Trends Plant Sci..

[36] Chen T.H., Murata N. (2002). Enhancement of tolerance of abiotic stress by metabolic engineering of betaines and other compatible solutes. Curr. Opin. Plant Biol..

[37] Chen T.H., Murata N. (2008). Glycinebetaine: An effective protectant against abiotic stress in plants. Trends Plant Sci..

[38] Liu Y., Zhang D., Xu Y., Yi Y. (2024). How the xerophytic moss Pogonatum inflexum tolerates desiccation. Plant Cell Rep..

[39] Asami P., Rupasinghe T., Moghaddam L., Njaci I., Roessner U., Mundree S., Williams B. (2019). Roots of the resurrection plant *Tripogon loliiformis* survive desiccation without the activation of autophagy pathways by maintaining energy reserves. Front. Plant Sci..

[40] Dai T., Ban S., Han L., Li L., Zhang Y., Zhang Y., Zhu W. (2024). Effects of exogenous glycine betaine on growth and development of tomato seedlings under cold stress. Front. Plant Sci..

[41] Wang X., Chen S., Zhang H., Shi L., Cao F., Guo L., Xie Y., Wang T., Yan X., Dai S. (2010). Desiccation tolerance mechanism in resurrection fern-ally *Selaginella tamariscina* revealed by physiological and proteomic analysis. J. Proteome Res..

[42] Pandey V., Ranjan S., Deeba F., Pandey A.K., Singh R., Shirke P.A., Pathre U.V. (2010). Desiccation-induced physiological and biochemical changes in resurrection plant, *Selaginella bryopteris*. J. Plant Physiol..

[43] Shivaraj Y.N., Plancot B., Ramdani Y., Gügi B., Kambalagere Y., Jogaiah S., Driouich A., Govind S.R. (2021). Physiological and biochemical responses involved in vegetative desiccation tolerance of resurrection plant *Selaginella brachystachya*. 3 Biotech.

[44] Li A., Wang D., Yu B., Yu X., Li W. (2014). Maintenance or collapse: Responses of extraplastidic membrane lipid composition to desiccation in the resurrection plant *Paraisometrum mileense*. PLoS ONE.

[45] Flores-Molina M.R., Thomas D., Lovazzano C., Núnez A., Zapata J., Kumar M., Correa J.A., Contreras-Porcia L. (2014). Desiccation stress in intertidal seaweeds: Effects on morphology, antioxidant responses and photosynthetic performance. Aquat. Bot..

[46] Kumar M., Gupta V., Trivedi N., Kumari P., Bijo A., Reddy C., Jha B. (2011). Desiccation induced oxidative stress and its biochemical responses in intertidal red alga *Gracilaria corticata* (Gracilariales, Rhodophyta). Environ. Exp. Bot..

[47] Contreras-Porcia L., Thomas D., Flores V., Correa J.A. (2011). Tolerance to oxidative stress induced by desiccation in *Porphyra columbina* (Bangiales, Rhodophyta). J. Exp. Bot..

[48] Foyer C.H., Noctor G. (2005). Redox homeostasis and antioxidant signaling: A metabolic interface between stress perception and physiological responses. Plant Cell.

[49] Wu X., Gu L., Prior R.L., McKay S. (2004). Characterization of anthocyanins and proanthocyanidins in some cultivars of *Ribes*, *Aronia*, and *Sambucus* and their antioxidant capacity. J. Agric. Food Chem..

[50] Singh R., Kaushik S., Wang Y., Xiang Y., Novak I., Komatsu M., Tanaka K., Cuervo A.M., Czaja M.J. (2009). Autophagy regulates lipid metabolism. Nature.

[51] Mi C., Wang Q., Zhao Y., Zhang C., Sun C., Liu Z., Lin L. (2022). Changes in the differentially expressed proteins and total fatty acid contents in winter rapeseed (*Brassica rapa* L.) leaves under drought stress. Russ. J. Plant Physiol..

[52] Bettaieb I., Zakhama N., Wannes W.A., Kchouk M., Marzouk B. (2009). Water deficit effects on *Salvia officinalis* fatty acids and essential oils composition. Sci. Hortic..

[53] Fujii S., Uenaka M., Nakayama S., Yamamoto R., Mantani S. (2001). Effects of sodium chloride on the fatty acids composition in *Boekelovia hooglandii* (Ochromonadales, Chrysophyceae). Phycol. Res..

[54] Xu L., Han L., Huang B. (2011). Membrane fatty acid composition and saturation levels associated with leaf dehydration tolerance and post-drought rehydration in *Kentucky bluegrass*. Crop Sci..

[55] Miao X., Zhang L., Chen X., Wu S., Niu D., FU H. (2015). The relationship of fatty acid composition and resistance of *Artemisia sphaerocephala* seedlings under water stress. Acta Prataculturae Sin..

[56] Liu X., Huang B. (2004). Changes in fatty acid composition and saturation in leaves and roots of creeping bentgrass exposed to high soil temperature. J. Am. Soc. Hortic. Sci..

[57] Pham Thi A.T., Vieira Da Silva J., Mazliak P. (1990). The role of membrane lipids in drought resistance of plants. Bull. Société Bot. France. Actual. Bot..

[58] Yin L., Xu J., Zhang L., Liu D., Zhang C., Liu T., Wang S., Deng X. (2024). Altered fatty acid composition confers improved drought acclimation in maize. Plant Physiol. Biochem..

[59] Chen D., Wang S., Qi L., Yin L., Deng X. (2018). Galactolipid remodeling is involved in drought-induced leaf senescence in maize. Environ. Exp. Bot..

[60] Liu X., Ma D., Zhang Z., Wang S., Du S., Deng X., Yin L. (2019). Plant lipid remodeling in response to abiotic stresses. Environ. Exp. Bot..

[61] Zorin B., Pal-Nath D., Lukyanov A., Smolskaya S., Kolusheva S., Didi-Cohen S., Boussiba S., Cohen Z., Khozin-Goldberg I., Solovchenko A. (2017). Arachidonic acid is important for efficient use of light by the microalga *Lobosphaera incisa* under chilling stress. Biochim. Biophys. Acta (BBA)-Mol. Cell Biol. Lipids.

[62] Du B., Kruse J., Winkler J.B., Alfarraj S., Albasher G., Schnitzler J.-P., Ache P., Hedrich R., Rennenberg H. (2021). Metabolic responses of date palm (*Phoenix dactylifera L*.) leaves to drought differ in summer and winter climate. Tree Physiol..

[63] Savchenko T., Walley J.W., Chehab E.W., Xiao Y., Kaspi R., Pye M.F., Mohamed M.E., Lazarus C.M., Bostock R.M., Dehesh K. (2010). Arachidonic acid: An evolutionarily conserved signaling molecule modulates plant stress signaling networks. Plant Cell.

[64] Ghassemi-Golezani K., Farhangi-Abriz S. (2021). Plant responses to exogenous salicylic and jasmonic acids under drought stress. Jasmonates Salicylates Signal. Plants.

[65] Abouelsaad I., Renault S. (2018). Enhanced oxidative stress in the jasmonic acid-deficient tomato mutant def-1 exposed to NaCl stress. J. Plant Physiol..

[66] Kaur G., Tak Y., Asthir B. (2022). Salicylic acid: A key signal molecule ameliorating plant stresses. Cereal Res. Commun..

[67] Liu A., Wang M., Dong J., Yan Z., Wang X., Li J., Song H. (2024). Foliar application of exogenous salicylic acid mitigates the detrimental effects caused by salt stress in sunflower seedlings. Ind. Crops Prod..

[68] Hedden P., Thomas S.G. (2012). Gibberellin biosynthesis and its regulation. Biochem. J..

[69] Hedden P., Phillips A.L. (2000). Gibberellin metabolism: New insights revealed by the genes. Trends Plant Sci..

[70] Colebrook E.H., Thomas S.G., Phillips A.L., Hedden P. (2014). The role of gibberellin signalling in plant responses to abiotic stress. J. Exp. Biol..

[71] Chen H.-I., Li P.-F., Yang C.-H. (2019). NAC-like gene GIBBERELLIN SUPPRESSING FACTOR regulates the gibberellin metabolic pathway in response to cold and drought stresses in *Arabidopsis*. Sci. Rep..

[72] Shohat H., Cheriker H., Kilambi H.V., Illouz Eliaz N., Blum S., Amsellem Z., Tarkowská D., Aharoni A., Eshed Y., Weiss D. (2021). Inhibition of gibberellin accumulation by water deficiency promotes fast and long-term ‘drought avoidance’ responses in tomato. New Phytol..

[73] Liu F., Xing S., Ma H., Du Z., Ma B. (2013). Cytokinin-producing, plant growth-promoting rhizobacteria that confer resistance to drought stress in *Platycladus orientalis* container seedlings. Appl. Microbiol. Biotechnol..

[74] Zhao Y. (2010). Auxin biosynthesis and its role in plant development. Annu. Rev. Plant Biol..

[75] Vishwakarma K., Upadhyay N., Kumar N., Yadav G., Singh J., Mishra R.K., Kumar V., Verma R., Upadhyay R., Pandey M. (2017). Abscisic acid signaling and abiotic stress tolerance in plants: A review on current knowledge and future prospects. Front. Plant Sci..

[76] Zhang C., Chen J., Yang R., Luo Q., Wang T., Zhang P., Chen H. (2022). Abscisic acid activates desiccation tolerance responses in intertidal seaweed *Neoporphyra haitanensis*. Front. Mar. Sci..

[77] Yoshida T., Christmann A., Yamaguchi-Shinozaki K., Grill E., Fernie A.R. (2019). Revisiting the basal role of ABA–roles outside of stress. Trends Plant Sci..

[78] Avramova Z. (2019). Defence-related priming and responses to recurring drought: Two manifestations of plant transcriptional memory mediated by the ABA and JA signalling pathways. Plant Cell Environ..

[79] Chen C., Ji D., Xie C., Xu Y., Liang Y., Zheng Y., Shi X., Wang F., Zhao L. (2008). Preliminary study on selecting the high temperature resistance strains and economic traits of *Porphyra haitanensis*. Acta Oceanol. Sin..

[80] Tukozkan N., Erdamar H., Seven I. (2006). Measurement of total malondialdehyde in plasma and tissues by high-performance liquid chromatography and thiobarbituric acid assay. Firat Tip Derg..

[81] Zhang C., Yi X., Gao X., Wang M., Shao C., Lv Z., Chen J., Liu Z., Shen C. (2020). Physiological and biochemical responses of tea seedlings (*Camellia sinensis*) to simulated acid rain conditions. Ecotoxicol. Environ. Saf..

[82] Wang Y.-T., Chen Z.-Y., Jiang Y., Duan B.-B., Xi Z.-M. (2019). Involvement of ABA and antioxidant system in brassinosteroid-induced water stress tolerance of grapevine (*Vitis vinifera* L.). Sci. Hortic..

[83] Dunn W.B., Broadhurst D., Begley P., Zelena E., Francis-McIntyre S., Anderson N., Brown M., Knowles J.D., Halsall A., Haselden J.N. (2011). Procedures for large-scale metabolic profiling of serum and plasma using gas chromatography and liquid chromatography coupled to mass spectrometry. Nat. Protoc..

[84] Wen B., Mei Z., Zeng C., Liu S. (2017). metaX: A flexible and comprehensive software for processing metabolomics data. BMC Bioinform..

[85] Li Y., Beisson F., Pollard M., Ohlrogge J. (2006). Oil content of *Arabidopsis* seeds: The influence of seed anatomy, light and plant-to-plant variation. Phytochemistry.

[86] Floková K., Tarkowská D., Miersch O., Strnad M., Wasternack C., Novák O. (2014). UHPLC–MS/MS based target profiling of stress-induced phytohormones. Phytochemistry.

