# Supplementary figures and images for: Metabolic Responses of Pyropia haitanensis to Dehydration-Rehydration Cycles Revealed by Metabolomics
Source: Mar Drugs. 2025 May 8;23(5):203. doi: 10.3390/md23050203 (PMC12113544; doi:10.3390/md23050203)

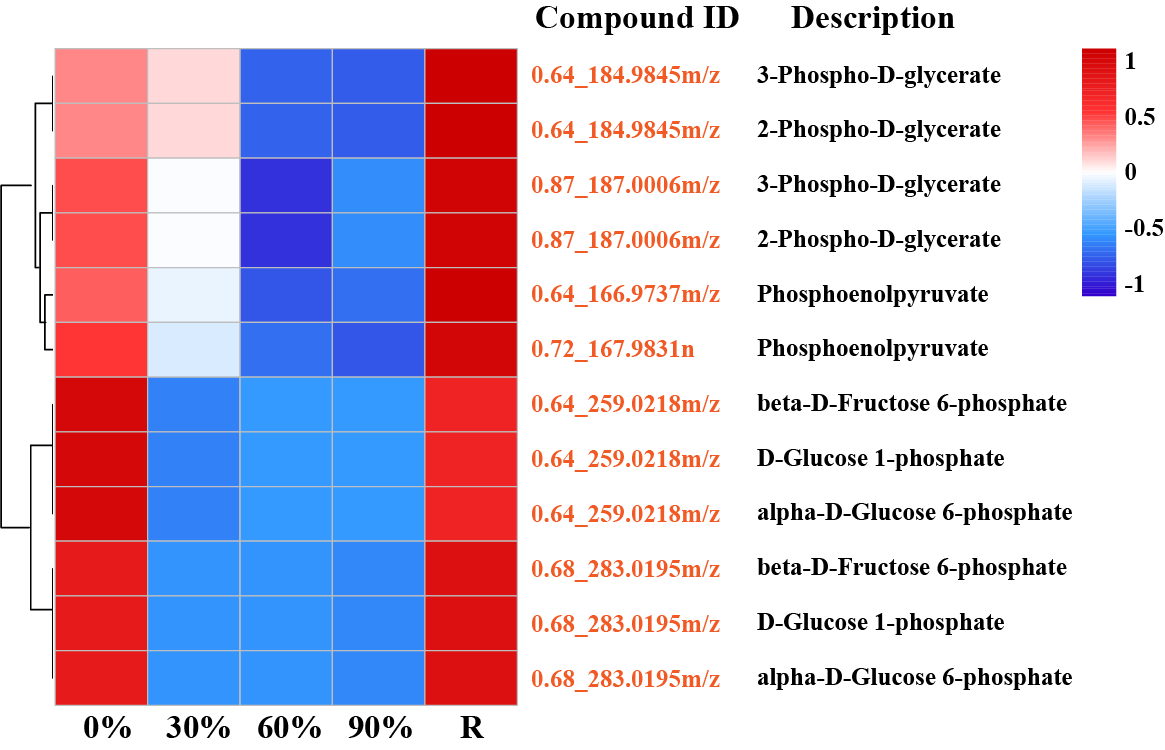

Supplement: Supplementary file 1 [file marinedrugs-23-00203-s001.zip › Supplementary Figure S1.tif]

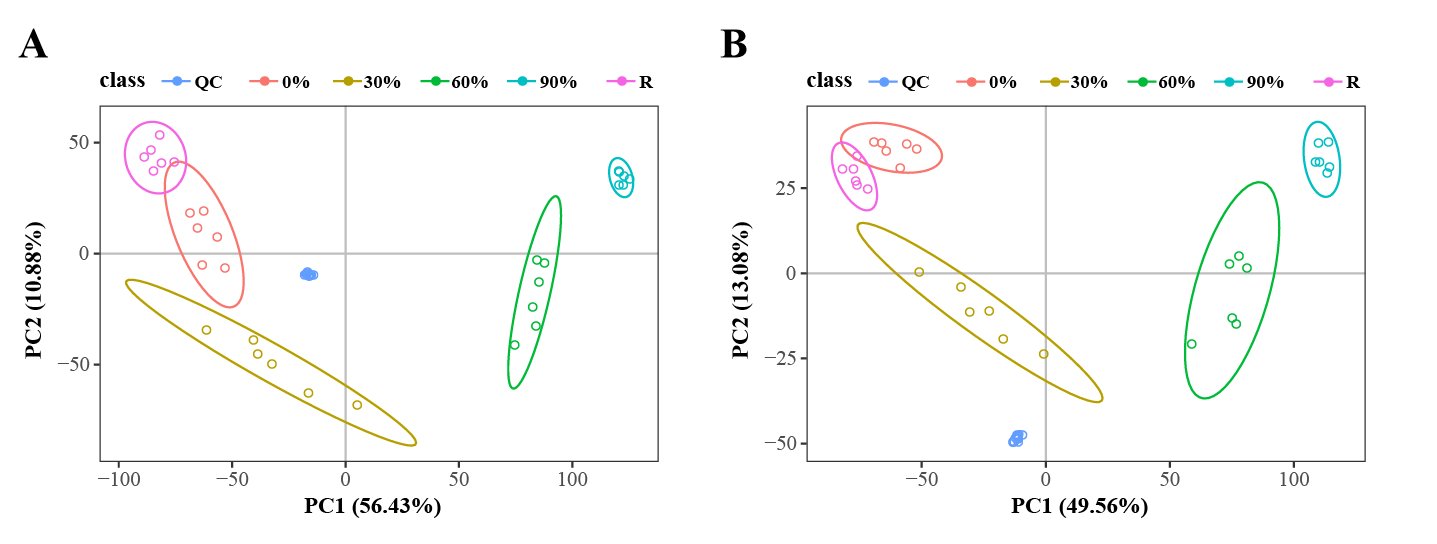

Supplement: Supplementary file 1 [file marinedrugs-23-00203-s001.zip › Supplementary Figure S2.tif]
